# Analysis of URI Nuclear Interaction with RPB5 and Components of the R2TP/Prefoldin-Like Complex

**DOI:** 10.1371/journal.pone.0063879

**Published:** 2013-05-08

**Authors:** Paolo Mita, Jeffrey N. Savas, Susan Ha, Nabil Djouder, John R. Yates, Susan K. Logan

**Affiliations:** 1 Department of Biochemistry and Molecular Pharmacology, New York University School of Medicine, New York, New York, United States of America; 2 Department of Chemical Physiology, The Scripps Research Institute-CA, La Jolla, California, United States of America; 3 Department of Urology, New York University School of Medicine, New York, New York, United States of America; 4 Centro Nacional de Investigaciones Oncológicas, CNIO, Fundación Banco Bilbao Vizcaya (F-BBVA)-CNIO Cancer Cell Biology Programme, Madrid, Spain; University of Geneva, Switzerland

## Abstract

Unconventional prefoldin RPB5 Interactor (URI) was identified as a transcriptional repressor that binds RNA polymerase II (pol II) through interaction with the RPB5/POLR2E subunit. Despite the fact that many other proteins involved in transcription regulation have been shown to interact with URI, its nuclear function still remains elusive. Previous mass spectrometry analyses reported that URI is part of a novel protein complex called R2TP/prefoldin-like complex responsible for the cytoplasmic assembly of RNA polymerase II. We performed a mass spectrometry (MS)-based proteomic analysis to identify nuclear proteins interacting with URI in prostate cells. We identified all the components of the R2TP/prefoldin-like complex as nuclear URI interactors and we showed that URI binds and regulates RPB5 protein stability and transcription. Moreover, we validated the interaction of URI to the P53 and DNA damage-Regulated Gene 1 (PDRG1) and show that PDRG1 protein is also stabilized by URI binding. We present data demonstrating that URI nuclear/cytoplasmic shuttling is affected by compounds that stall pol II on the DNA (α-amanitin and actinomycin-D) and by leptomycin B, an inhibitor of the CRM1 exportin that mediates the nuclear export of pol II subunits. These data suggest that URI, and probably the entire R2TP/prefoldin-like complex is exported from the nucleus through CRM1. Finally we identified putative URI sites of phosphorylation and acetylation and confirmed URI sites of post-transcriptional modification identified in previous large-scale analyses the importance of which is largely unknown. However URI post-transcriptional modification was shown to be essential for URI function and therefore characterization of novel sites of URI modification will be important to the understanding of URI function.

## Introduction

The Unconventional prefoldin RPB5 Interactor (URI), also called RMP (RPB5-mediating protein), was identified as a transcriptional repressor that interacts with the RPB5/POLR2E subunit of the three RNA polymerases [1]. In the mitochondria URI was shown to bind and regulate the phosphatase PP1γ, thereby controlling apoptosis downstream of the mToR pathway [2]. Interestingly, recent studies demonstrate that URI is an oncogene involved in the development of ovarian cancer [3] and hepatocarcinoma [4]. We previously showed that, in prostate cells, URI behaves as an androgen receptor co-repressor in complex with the androgen receptor trapped clone-27 (Art-27) [5]. The two proteins interact through their prefoldin alpha domains and affect each other's stability. Both URI and Art-27 are prefoldin-like proteins that have high homology with the components of the heterohexameric co-chaperone Prefoldin complex, which is responsible for the presentation of unfolded proteins, such as actin, to the chaperonin complex CCT/c-cpn/TRiC [6]. A mass spectrometry analysis revealed URI and Art-27 to be part of a complex called R2TP/Prefoldin-like complex composed of 4 subunits in common with the yeast R2TP complex (RPAP3, Pih1D1, RUVBL1 and RUVBL2) and of 5 prefoldin or prefoldin-like proteins (URI, Art-27, PDRG1, PFD2 and PFD6) [7]. The R2TP/prefoldin-like complex, together with Hsp90, is responsible for the assembly of the RNA polymerase II complex (pol II) in the cytoplasm of eukaryotic cells. Also, a prediction of previously performed mass spectrometry analyses indicates that there is a URI/prefoldin complex that includes URI, the two ATP-dependent chromatin modifiers RUVBL1/Tip49 and RUVBL2/TIP48, Art-27/UXT1, RPB5, PDRG1 and PFD2 [8]. An independent analysis of Art-27/STAP1 interacting proteins in HeLa cells also identified URI, PFDN2, RUVBL1 and RUVBL2 as interacting proteins [9] suggesting that these proteins are part of a stable multiprotein complex. Despite the numerous proteomic analyses confirming the existence of the URI/prefoldin complex, few and scattered experimental data validate or deepen the understanding of the R2TP/prefoldin-like complex that is proposed to be responsible for the fundamental process of assembling the RNA polymerase II complex. We used a mass spectrometry (MS) approach to conduct an unbiased screen of URI nuclear interactors in prostate cells and confirmed the interaction of URI with subunits of the R2TP/prefoldin-like complex. Interestingly, URI protein affects the stability of several components of the complex including RPB5 and PDRG1 as we previously showed for Art-27 [5]. Our data support the idea that URI and Art-27 translocate from the nucleus to the cytoplasm through the exportin CRM1 which is also used by RNA polymerase subunits. Our MS analysis also identified several URI novel sites of phosphorylation and acetylation that we show do not affect RPB5 or Art-27 binding. Overall our work advances the understanding and characterization of the R2TP/prefoldin-like complex and sheds light on the interplay of components of this newly identified biologically fundamental complex.

## Materials and Methods

### Cell culture and stable cell lines

Cells were cultured as previously reported in [5]. LNCaP cells stably overexpressing the FLAG-URI alpha construct or an empty vector as well as LNCaP stable cell lines overexpressing a non silencing shRNA (shNS) or a shRNA against URI (shURI) were previously described in [5]. LNCaP cells stably overexpressing a FLAG-URI-EGFP were generated by Lipofectamine 2000 transfection followed by selection with geneticin (0.5 mg/ml). After selection cells were FACS-sorted to eliminate the EGFP non-expressing cells.

### Overexpression, luciferase assay and siRNA transfection

All tranfection experiments were conducted transfecting a total of 10 µg of DNA in 293 or LNCaP cells. Lipofectamine or Lipofectamine 2000 (Invitrogen) were used for transfection in 293HEK or LNCaP cells, respectively. Luciferase assay and siRNA transient transfection were performed as described in [5]. The constructs used are as follows: PDRG1 in pCMV6-XL5 (Origene SC112755), POLR2E in pCMV6-AC (Origene SC319449), URI alpha previously used in [5].

### qPCR

Total RNA was isolated using Trizol Reagent (Ambion). Total RNA was reverse transcribed at 55°C for 1 h using Superscript III reverse transcriptase and oligo(dT)20 primers (Invitrogen). Real-time PCR was performed using gene-specific primers and 2×SYBR green Taq-ready mix (Sigma-Aldrich). Data were analyzed by the ΔΔCt method using RPL19 as a control gene and were normalized to the values for control samples, which were arbitrarily set to 1. The sequences of the primers used for qPCR analysis are as follows:

RPB5-F: CGCGCTACTTTGGGATAAAG;

RPB5-R: ACCAGCCGGTAGGTGATGTA;

URI-F: CACCTGAGGCTTTTTCTGGA;

URI-R: GGGGTGGTGTTAAGGAAGGT;

PDRG1-F: GAAAAACTGCGGAAGCAACT;

PDRG1-R: CCCCATCTTGGTTCTTGAGT;

RUVBL1-F: ATTGGCACCAAGACCACACT;

RUVBL1-R: CCTTCCCGTTGATTTTAGCA;

RUVBL2-F: AGGACGCCTTCCTCTTCAAC;

RUVBL2-R: CCAACTCAGGAGGTGTCCAT;

### Nuclear-cytoplasmic fractionation and western blotting

For the isolation of cytoplasmic and nuclear fractions cells were scraped in PBS and resuspended in one packed cell volume of hypotonic 1× Buffer A (10× Buffer A: 100 mM Hepes, 15 mM MgCl2, 100 mM KCl; solution pH 7.9). Cells were left swelling in buffer A for 15 minutes on ice and then passed through a 23–26 gauge syringe needle. The solution was then centrifuged at 17,000×g for 6 minutes at 4°C. The supernatant (cytoplasmic fraction) was spun again at 17,000×g for 15 minutes while the pellet was resuspended in one pellet volume of Buffer B (10 mM Hepes, 25% glycerol, 420 mM NaCl, 1.5 mM MgCl2, 0.2 mM EDTA; solution pH 7.9). Nuclei solution was incubated for 30 minutes on ice while stirring and then spun at 17,000×g for 6 minutes. The supernatant (nuclear extract) was collected and the salt concentration adjusted to 300 mM using HEMG0 buffer (25 mM Hepes, 12.5 mM MgCl2, 10% glycerol, 1 mM EDTA). Triton X-100 was added to a final concentration of 1% and, after 30 minutes incubation on ice, the solution was centrifuged at 17,000×g for 15 minutes. The supernatant was then used for immunoprecipitation experiments.

Western blot analysis of RUVBL1, RUVBL2 and RPB5 co-immunoprecipiation with URI were performed using rabbit or mouse ONE-HOUR Complete IP-Western kits (GenScript) as described by the company. These kits eliminate the light and heavy chain signal produced by the antibodies used for immunoprecipiatetion. All the other Western blots were performed by transferring the proteins onto Immobilon-P membrane (Fisher), blocking with TBS+5% BSA (blocking solution) and incubating the membrane in primary antibody solubilized in blocking solution.

### Mass spectrometry analysis and immunoprecipitation

Sample Preparation: 15×10^8^ LNCaP-vector and LNCaP-FLAG-URI cells were used to isolate nuclear extracts as described above. FLAG-URI was immunoprecipitated from the nuclear extracts using FLAG-conjugated agarose beads (SIGMA A2220). After 4 hours incubation with the samples, the beads were washed 3 times in HEMG300 (HEMG buffer with 300 mM KCl), 3 times in HEMG150 (HEMG buffer with 150 mM KCl) and twice in TBS. After the last wash beads were resuspended in 35 µl of TBS and 3 µl of 3×FLAG peptide 5 mg/ml (SIGMA F4799) were added. The solutions were incubated for 1 hour at 4°C, tapping the tubes every 10 minutes to resuspend the beads. The supernatant containing the URI interacting proteins was collected and proteins precipitated with Trichloroacetic acid (TCA). URI interacting proteins and proteins immunoprecipitated from LNCaP-vect. cells were precipitated with Trichloroacetic acid (TCA). TCA precipitate was resuspended in 8 M urea. Next the extracts were processed with ProteasMAX (Promega, Madison, WI, USA) per the manufacturer's instruction. The samples were subsequently reduced by 20 minute incubation with 5 mM TCEP (tris(2 carboxyethyl)phosphine) at room temperature and alkylated in the dark by treatment with 10 mM Iodoacetamide for 20 additional minutes. The proteins were digested over-night at 37 degrees with Sequencing Grade Modified Trypsin (Promega, Madison, WI, USA) and the reaction was stopped by acidification with formic acid. Multidimensional Protein Identification Technology (MudPIT) and LTQ and LTQ Orbitrap Mass Spectromtery: The protein digest was pressure-loaded onto a 250 µm i.d capillary packed with 2.5 cm of 10-µm Jupiter C18 resin (Phenomenex, Torrance, CA, USA) followed by an additional 2.5 cm of 5-µm Partisphere strong cation exchanger (Whatman, Clifton, NJ). The column was washed with buffer containing 95% water, 5% acetonitrile, and 0.1% formic acid. After washing, a 100-µm i.d capillary with a 5-µm pulled tip packed with 15 cm 4-µm Jupiter C18 resin (Phenomenex, Torrance, CA, USA) was attached to the filter union and the entire split-column (desalting column–filter union–analytical column) was placed in line with an Agilent 1100 quaternary HPLC (Palo Alto, CA) and analyzed using a modified 5-step separation described previously (Washburn et al., 2001). The buffer solutions used were 5% acetonitrile/0.1% formic acid (buffer A), 80% acetonitrile/0.1% formic acid (buffer B), and 500 mM ammonium acetate/5% acetonitrile/0.1% formic acid (buffer C). Step 1 consisted of a 75 min gradient from 0–100% buffer B. Steps 2–5 had a similar profile except 3 min of 100% buffer A, 5 min of X% buffer C, a 10 min gradient from 0–15% buffer B, and a 105 min gradient from 10–55% buffer B (except for step 5 which %B was increased from 10% to 100%). The 5 min buffer C percentages (X) were 10, 40, 60, 100% respectively for the 5-step analysis. As peptides eluted from the microcapillary column, they were electrosprayed directly into an LTQ mass spectrometer (ThermoFinnigan, Palo Alto, CA). For LTQ analysis: As peptides eluted from the microcapillary column, they were electrosprayed directly into an LTQ 2-dimensional ion trap mass spectrometer (ThermoFinnigan, Palo Alto, CA) with the application of a distal 2.4 kV spray voltage. A cycle of one full-scan mass spectrum (400–2000 m/z) followed by 7 data-dependent MS/MS spectra at a 35% normalized collision energy was repeated continuously throughout each step of the multidimensional separation. Application of mass spectrometer scan functions and HPLC solvent gradients were controlled by the Xcalibur datasystem. Analysis of Tandem Mass Spectra: Protein identification and quantification analysis were done with Integrated Proteomics Pipeline (IP2, Integrated Proteomics Applications, Inc. San Diego, CA) using ProLuCID, DTASelect2 and Census. Tandem mass spectra were extracted into ms1 and ms2 files [10] from raw files using RawExtract 1.9.9 (http://fields.scripps.edu/downloads.php) and were searched against IPI human protein database (version 3_57_01, released on 01-01-2009; plus sequences of known contaminants such as keratin and porcine trypsin concatenated to a decoy database in which the sequence for each entry in the original database was reversed [11]using ProLuCID/Sequest [12]. LTQ data was searched with 3000.0 milli-amu precursor tolerance and the fragment ions were restricted to a 600.0 ppm tolerance. All searches were parallelized [13] and performed on The Scripps Research Institute's garibaldi 64-bit LINUX cluster with 2848 cores. Search space included all fully- and half-tryptic peptide candidates with no missed cleavage restrictions. Carbamidomethylation (+57.02146) of cysteine was considered a static modification and we require 2 peptides per protein and at least one trypitic terminus for each peptide identification. The ProLuCID search results were assembled and filtered using the DTASelect program (version 2.0) [14], [15] with false discovery rate (FDR) of 0.05; under such filtering conditions, the estimated false discovery rate was below ∼1% at the protein level in all analysis.

### Subcloning

All the URI deletions and truncations were generated using a pcDNA3-FLAG-URI construct previously used in [5]. Deletions were generated by PCR using specific primers with flanking XhoI restriction sequences. Therefore URI deletions have a unique XhoI restriction site at the point of deletion. Truncations were also generated by PCR using specific primers with a XbaI restriction sequence. XbaI was chosen because it was a unique restriction site present just downstream of the URI STOP codon. The FLAG tag was maintained in all the deletions and truncations. The generated truncations of wild type URI are the following: URIΔ150 is a URI deleted of aa 151–535 and maintains just the prefoldin-like domain, URIΔ270 is a URI deleted of aa 271–535 that maintains the prefoldin-like and the RPB5 binding domains, URIΔ340 is a URI deleted of aa 341–535 that maintains the prefoldin-like domain, the RPB5 binding domain and the asparagine rich domain. The generated deletions are the following: URIΔprefoldin is a URI deleted of aa 11–153 encompassing the entire prefoldin-like domain, URIΔhook is a URI deleted of aa 85–100 encoding 2 of the 4 beta strands of the prefoldin-like domain, URIΔRPB5 is a URI deleted of aa 177–224 encompassing the essential domain for the binding of RPB5. All constructs were confirmed by sequencing. EGFP-URI was generated by PCR reaction of the C terminus of FLAG-URI introducing a XhoI site and deleting the stop codon. The newly created FLAG-URI-XhoI was indroduced into a pcDNA3-EGFP construct (Addgene, plasmid 13031) using BamH1 and XhoI restriction sites.

### Immunofluorescence

LNCaP-URI-EGFP stable cell lines were plated on fibronectin coated glass coverslips. The next day cells were treated with 10 µg/ml α-amanitin, 10 µg/ml α-amanitin plus 15 nM leptomycin B or 5 µg/ml of actinomycin D for 12 hrs. Cells were then fixed in 4% formalin for 10 minutes and mounted on glass slides using mounting media with DAPI (Vectashield with DAPI, Vector laboratories). Pictures of FITC and DAPI fluorescence were taken using a Zeiss Axioplan Wide-field fluorescence/DIC upright microscope.

## Results

### URI is part of the R2TP/prefoldin-like complex

URI/RMP protein was identified as a transcriptional repressor that binds the RPB5 subunit of the three RNA polymerases [1]. Despite this observation the nuclear function of URI and the mechanism by which URI inhibits gene transcription remain elusive. To identify the components of this nuclear complex we performed an unbiased mass spectrometry (MS) analysis of nuclear URI interactors in LNCaP prostate cells. We immunoprecipitated URI from stable cell lines overexpressing FLAG tagged URI or from LNCaP control cells stably overexpressing an empty vector. We performed this analysis twice and identified several proteins that specifically coimmunoprecipitated with URI in both the replicate experiments. As expected, URI/RMP was the most abundant immunoprecipitated protein. Among the URI interactors we identified RPB5, Art-27 and all the other subunits of the R2TP/prefoldin-like complex (table 1). In one of the two MS-analysis replicates we retrieved RUVBL (RUVBL1 and RUVBL2) peptides from control cells, although the number of peptides immunoprecipitated from URI overexpressing cells was much greater compared to the peptides retrieved in the control immunoprecipitations indicating that, despite the low level non-specific binding, our analysis infers specific interaction of RUVBL1 and RUVBL2 proteins with URI.

**Table 1:** URI is part of the R2TP/prefoldin-like complex.

	Experiment 1		Experiment 2		
Accession	URI IP	CTRL IP	URI IP	CTRL IP	Description
IPI00477619	70	0	55	0	URI/RMP
IPI00002408	37	0	27	0	RPAP3
IPI00291093	23	0	18	0	RPB5/POLR2E
IPI00465211	22	0	18	0	WDR92
IPI00005657	19	0	15	0	PFDN6
IPI00006052	17	0	13	0	PFDN2
IPI00550995	15	0	13	0	PIH1D1
IPI00027887	14	0	13	0	PDRG1
IPI00170862	12	0	3	0	Art-27UXT
IPI00021187	18	5	18	0	RUVBL1
IPI00009104	18	2	21	0	RUVBL2

List of nuclear URI interactors obtained from immunoprecipitation of FLAG-URI from control cells (CTRL IP) and URI overexpressing cells (URI IP). The analysis was repeated twice and the number of peptides retrieved in the two analyses are reported together with the international protein index (Accession) and the name of each protein.

Of note, we did not identify DMAP1 (DNA methyltransferase 1 associated protein 1) as an URI interacting protein in prostate cells. It has been previously shown that in HLE cells (human hepatoma cell line) DMAP1 binds URI and dictates its nuclear translocation, possibly masking a cytoplasmic localization sequence in the second α-helix of the URI prefoldin-like domain [16]. We performed several experiments to explore the possible binding of URI to DMAP1 protein but we were not able to observe binding or colocalization between the two proteins (data not shown).

Overall our data validate and support the findings previously characterizing the R2TP/prefoldin-like complex [7]. It is interesting to note that we identify URI as a component of the R2TP/prefoldin-like complex from nuclear extracts of prostate cells suggesting that there is nuclear import of the entire R2TP/prefoldin-like complex along with URI and the RNA polymerase II.

### URI binds RPB5 protein and affects its stability

URI was initially identified by Far-Western as a RNA pol II subunit 5 interacting protein and therefore named RMP (RPB5 mediating protein). The region of interaction of URI and RPB5 was mapped to a domain encompassing amino acids 177–257 of the currently annotated URI1/URIα sequence (GI:19924159). More specifically aa 177–224 were shown to be essential for URI binding to RPB5, while aa 224–257 have an accessory role [1]. Also RPB5 was shown to be the subunit functioning as the interface between pol II and the R2TP/prefoldin-like complex responsible for the cytoplasmic assembly of RNA polymerase II [7].

**Figure 1:**
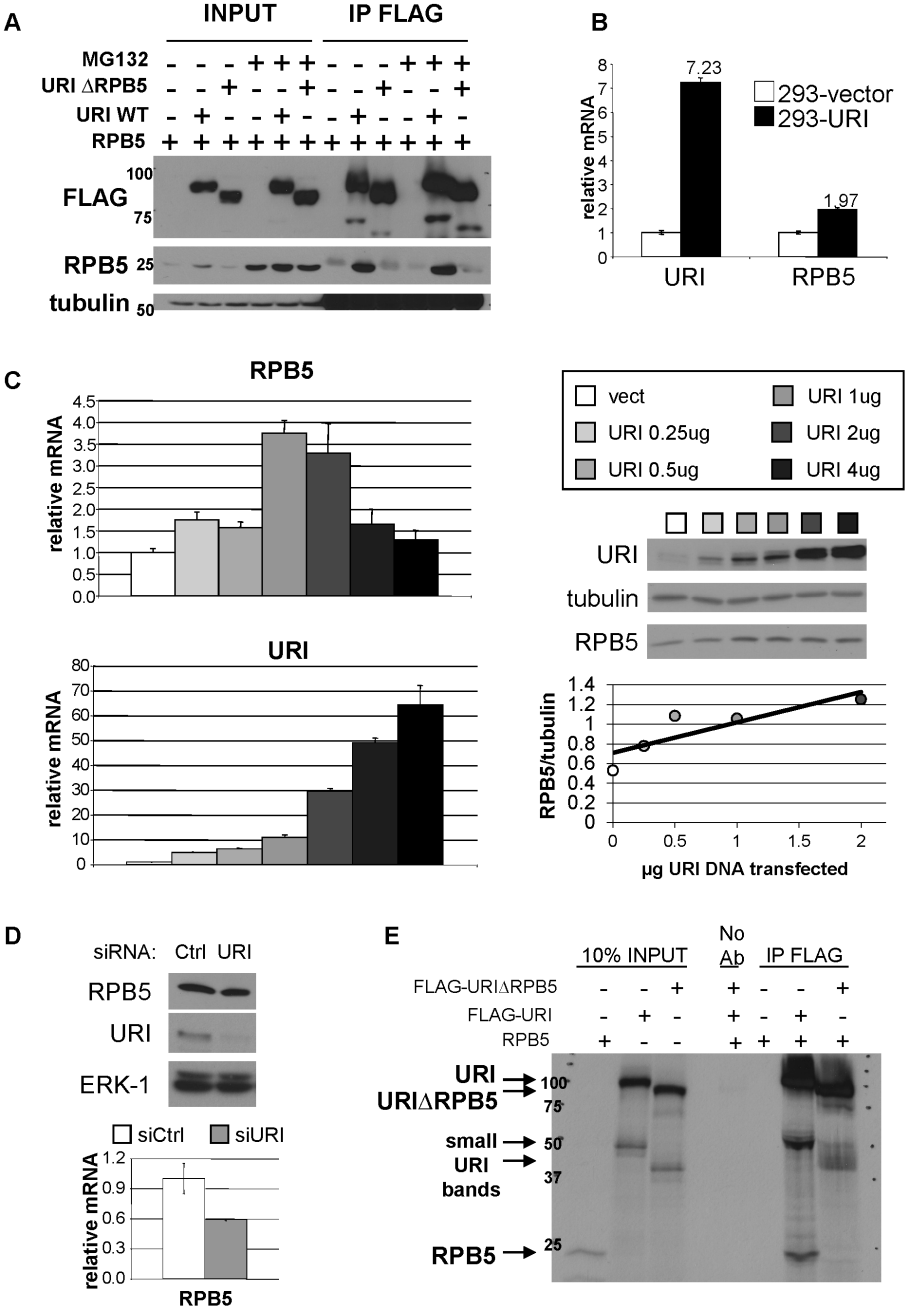
URI binds RPB5 protein and affects its protein stability and gene transcription. a) 293 cells were transfected with RPB5 and URI wild type (URI WT) or URI deleted of the RPB5 binding domain (URIΔRPB5). 48 hrs after transfection cells were lysed and part of the lysate used for the immunoprecipitation of URI using FLAG antibodies. Cells were either growth in the absence or presence of 25 µM MG132. The expression and immunoprecipiation of URI and RPB5 was analyzed by Western blotting. Tubulin was used as loading control. b) mRNA was isolated from 293 stable cell lines overexpressing an empty vector (293-vector) or a construct encoding FLAG-URI (293-URI). URI and RPB5 mRNA were quantified by qPCR. The URI and RPB5 mRNA of 293-vector cells were set as 1. c) The indicated µg of pcDNA3-FLAG-URI construct were transfected in LNCaP cells. 48 hours after transfection proteins and mRNA were isolated. RPB5 and URI mRNA and protein expression were measured by qPCR (left panels) and Western blot (right panels). RPB5 protein expression (quantified by densitometry analysis of the reported Western blot) is also plotted with the µg of transfected URI (R^2^ is 0.729). d) mRNA and proteins were isolated from LNCaP cells treated with control siRNA (siCtrl) or siRNA directed against URI (siURI). RPB5 mRNA and URI and RPB5 proteins were measured by qPCR (bottom) and Western blot (top). e) In vitro transcribed/translated and ^35^S labelled RPB5, URI WT and URIΔRPB5 proteins were mixed and used to immunoprecipitate URI with a FLAG antibody. Input solutions and immunoprecipitated complexes were analyzed by SDS-PAGE.

To validate our mass spectrometry analysis and better characterize the interaction of URI with RPB5 we subcloned a deleted FLAG-URI (URIΔRPB5) lacking the essential domain of interaction with RPB5 (aa 177–224). Immunoprecipitation of FLAG-URI from 293 cells overexpressing wild type FLAG-URI and RPB5 shows binding between the two proteins. As expected FLAG-URIΔRPB5 does not interact with RPB5 confirming the data previously reported by Dorisuren and colleagues [1]. Interestingly, overexpression of URI resulted in increased levels of RPB5 protein while overexpression of URIΔRPB5 did not have any effect on RPB5 expression (fig.1a, INPUT). This result suggests that URI binding to RPB5 is able to stabilize RPB5 protein in a similar manner to the previously reported URI-dependent stabilization of Art-27 [5]. To confirm that the absence of co-immunoprecipitation of URIΔRPB5 with RPB5 was due to absence of binding between the two proteins and not simply to a lower amount of RPB5 protein in cells overexpressing URIΔRPB5, we performed the immunoprecipitation assays in the presence of MG132. MG132 inhibits protein degradation through the proteasome and, therefore, we predicted it would increase the level of RPB5 protein even in cells not overexpressing URI. Indeed we observed that cells treated with MG132 have a similar elevated level of RPB5 protein. In these conditions RPB5 interacts with URI wild-type (WT) while it does not interact, or it interacts very weakly, with URIΔRPB5 (fig.1a). Also, the effect of URI overexpression on RPB5 gene transcription was analyzed by qPCR (fig.1b). mRNA was isolated from control HEK293 stable cell lines (293-vect) and from cells stably overexpressing URI (293-URI). Surprisingly, RPB5 mRNA was also increased in cells overexpressing URI. This result indicates that an elevated level of URI protein (about 7 times over control cells) not only stabilizes RPB5 protein but, directly or indirectly, induces an increase of RPB5 gene transcription (fig.1b). To further explore this unexpected effect of URI overexpression, increasing amounts of a URI encoding plasmid were transfected into LNCaP prostate cells and RPB5 mRNA and protein measured (fig.1c). Increasing expression of URI induced higher transcription of RPB5 up to a certain threshold of cellular URI. When URI mRNA was increased more than 30 times compared to control cells the URI-dependent effect on RPB5 transcription was no longer evident. In contrast, endogenous RPB5 protein levels were positively correlated (Spearman coefficient ρ = 0.9, p-value 0.037) with cellular URI (fig.1c, right panel). The increased protein levels of RBP5 with increasing URI levels suggest that the increase of RPB5 protein upon URI overexpression is primarily due to protein stabilization and only partly due to increased transcription. RPB5 protein and mRNA were also measured upon URI depletion by siRNA (fig.1d). As expected, depletion of URI also decreased RPB5 transcription (fig.1d, bottom panel). On the other hand we were unable to detect any effect of URI depletion on RPB5 protein (fig.1d, upper panel). It is possible that RPB5 is part of other non-URI containing multiprotein complexes within which the protein is stable, or perhaps cells are able to adapt to depleted levels of URI to sustain a constant level of RPB5 protein.

To demonstrate that URI and RPB5 also interact *in vitro* we transcribed/translated URI and RPB5 using an in vitro reticulocyte system. ^35^S was included in the reaction to label the proteins. A fraction of the in vitro transcribed/translated URI WT or URIΔRPB5 and RPB5 proteins were mixed 1∶1 and incubated at 4°C for 4 h. After incubation, FLAG-URI was immunoprecipiated using a FLAG antibody. The precipitated immunocomplexes were extensively washed and analyzed by SDS-PAGE. Reactions with no antibody or no URI construct were used as negative controls. Transcription/translation of URI and RPB5 produced proteins with the anticipated size (≈90 KDa for URI and ≈24 KDa for RPB5). Interestingly, translation of the URI constructs (both URI WT and URIΔRPB5) also produced smaller products that migrate between the 50 KDa and 37 KDa markers and were previously not described. RPB5 clearly immunoprecipiates with WT URI and not with URIΔRPB5. Also no bands were detected in the two negative control lanes (fig.1e). These observations confirm that URI and RPB5 bind each other through URI aa 177–224.

### URI-induced repression of androgen receptor mediated transcription does not require binding of URI to RPB5

**Figure 2:**
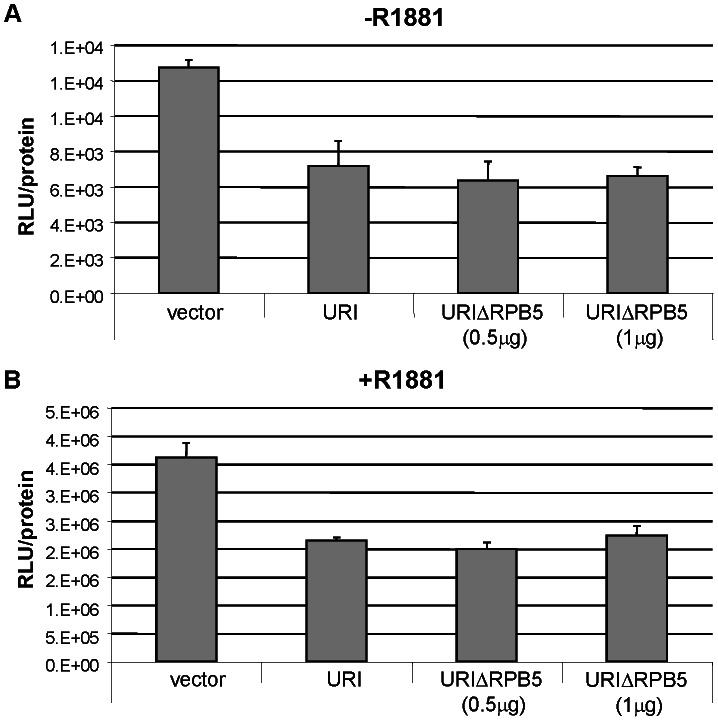
RPB5 binding is not necessary for URI mediated repression of AR mediated transcription. LB1-LNCaP cells were transfected with an empty vector, a vector encoding URI or URIΔRPB5. 0.5 µg or 1 µg of URIΔRPB5 construct were transfected. After 24 hrs of hormone starvation cells were treated with or without 10 nM R1881 for an additional 24 hrs. The luciferase units normalized by protein content are reported.

We previously reported that URI, as Art-27, behaves as an androgen receptor (AR) repressor [5]. We, therefore, performed luciferase experiments to explore the importance of RPB5 binding in the URI induced repression of AR-mediated transcription. LNCaP cells stably over-expressing a luciferase reporter downstream of the probasin promoter (LB1-LNCaP) containing known androgen response elements (AREs) were used to measure AR mediated transcription. An empty vector, a construct encoding wild type URI or a construct encoding URIΔRPB5 were transfected into LB1-LNCaP cells. Cells were treated for 24 hrs with or without 10 nM of the synthetic androgen R1881 and then luciferase activity was measured (fig.2). Both URI wild type and URIΔRPB5 reduce AR-mediated transcription of the luciferase reporter either in the absence (fig.2a) or presence (fig.2b) of R1881. This data suggests that URI-mediated repression of AR-dependent transcription does not occur by the binding of URI to RPB5 protein or perhaps to the entire RNA polymerase II complex.

### URI binds and stabilizes PDRG1 protein

P53 and DNA damage-Regulated Gene 1 (PDRG1) is a currently understudied gene that encodes a protein of approximately 15 KDa, characterized by a β-prefoldin-like domain [17]. PDRG1 gene transcription is upregulated by UV irradiation and downregulated by p53 protein. Interestingly PDRG1 protein expression was also shown to be increased in several cancers (colon, rectum, ovary, lung, stomach, breast and uterus) and depletion of PDRG1 in colon cancer cell lines was shown to induce a decrease in cell proliferation [18]. PDRG1 is part of the R2TP/prefoldin-like complex, which also comprises URI and Art-27 proteins [7]. According to our mass spectrometry analysis, PDRG1 is indeed a nuclear interactor of URI (table 1). PDRG1 has a molecular weight similar to Art-27, a protein that we showed binds strongly and directly to URI. Compared to Art-27, PDRG1 has an even more abundant number of peptides retrieved in our two immunoprecipitation/mass spectrometry experiments, suggesting an abundant interaction between URI and PDRG1.

**Figure 3:**
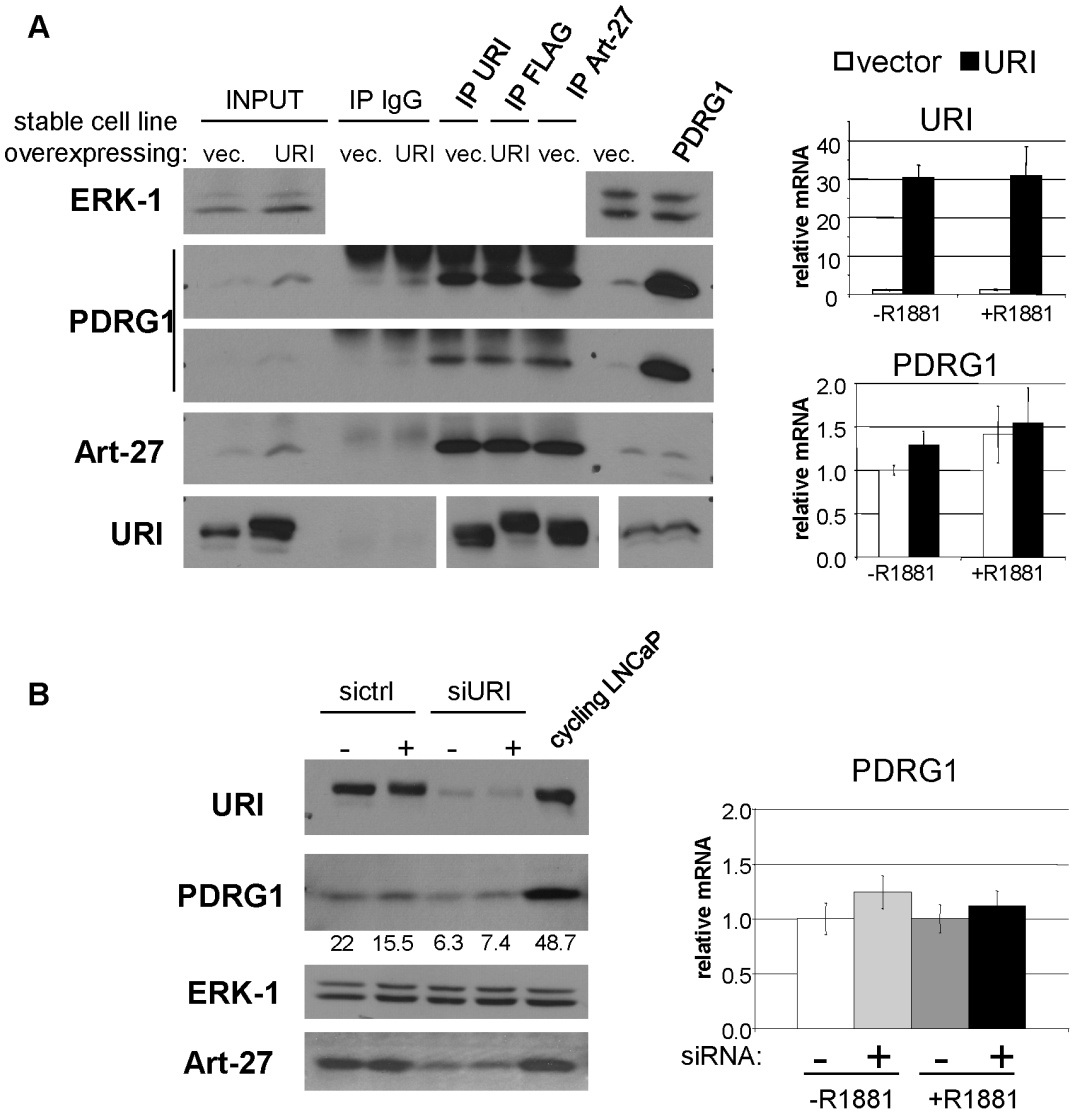
URI interacts with PDRG1. a) LNCaP-vector cells and LNCaP-URI stable cell lines were used to immunoprecipitate URI, FLAG-URI and Art-27. The immunocomplexes were analyzed by Western blotting using the indicated antibodies. ERK1 is used as loading control. LNCaP cells transiently overexpressing an empty vector or a construct encoding PDRG1 were also analyzed for the expression of PDRG1, URI and Art-27. URI and PDRG1 mRNA expression in control LNCaP cells (vector) and LNCaP cells overexpressing URI (URI) treated with or without synthetic androgen R1881 are reported on the right panels. b) URI was depleted from LNCaP cells by sequential siRNA transfection as described in materials and methods. After knock down cells were treated with or without 10 nM R1881 for 24 hrs and then lysed in Triton buffer or their mRNA was isolated. Cell lysates were analysed for Art-27, PDRG1 and URI expression by Western blotting and qPCR (right panel). ERK1 protein is used as loading control and LNCaP cell lysates from normally cycling cells cultured in complete media are used for comparison. The numbers reported below the PDRG1 blotting are densitometry analysis of PDRG1 protein bands (ImageJ).

Immunoprecipitation experiments validated the interaction of URI with PDRG1 in prostate cells. LNCaP cells stably expressing an empty vector or a construct encoding FLAG-URI were used to assay PDRG1 interaction with endogenous and over-expressed URI as well as with endogenous Art-27 (fig.3a). Lysates from LNCaP-vector and LNCaP-URI cells were used to immunoprecipitate endogenous and overexpressed URI with a monoclonal anti-URI antibody, overexpressed URI with a FLAG antibody and endogenous Art-27 with a polyclonal anti-Art-27 antibody. As expected, endogenous or overexpressed URI efficiently pulled down endogenous Art-27. Interestingly the amount of Art-27 protein coimmunoprecipitated with URI or FLAG-URI is the same as the amount of Art-27 protein immunoprecipitated using anti-Art-27 antibody, suggesting that the URI-Art-27 complex(es) are tightly bound in vivo. The same behavior was observed for PDRG1 protein which co-immunoprecipitates with overexpressed FLAG-URI, endogenous URI or with Art-27. Interestingly, we noticed that, as for RPB5 and Art-27, overexpression of URI induced increased PDRG1 protein (fig.3a, first two lanes). Overexpression of PDRG1 in LNCaP cells, on the other hand, does not seem to affect the endogenous Art-27 or URI proteins (fig.3a, last 2 lanes). To explore the effect of URI depletion on PDRG1 protein, lysates obtained from LNCaP cells depleted of URI by siRNA treatment were analyzed for PDRG1 and URI protein expression. Cells were also treated with or without 10 nM R1881 to assess if PDRG1 protein was affected by androgen treatment. Similar to Art-27, knock down of URI either in the presence or absence of hormone, induces a decrease of PDRG1 protein (fig.3b, left panel). Transcription of the PDRG1 gene in conditions of overexpressed (fig.3a, right panel) or depleted (fig.3b, right panel) URI was measured by qPCR. Contrary to the effect of URI on RPB5 transcription, URI overexpression as well as depletion did not affect PDRG1 mRNA, suggesting that the changes in PDRG1 proteins are exclusively due to post-transcriptional effects of URI protein on RPB5. Therefore, the binding of PDRG1 to URI likely stabilizes PDRG1 protein.

**Figure 4:**
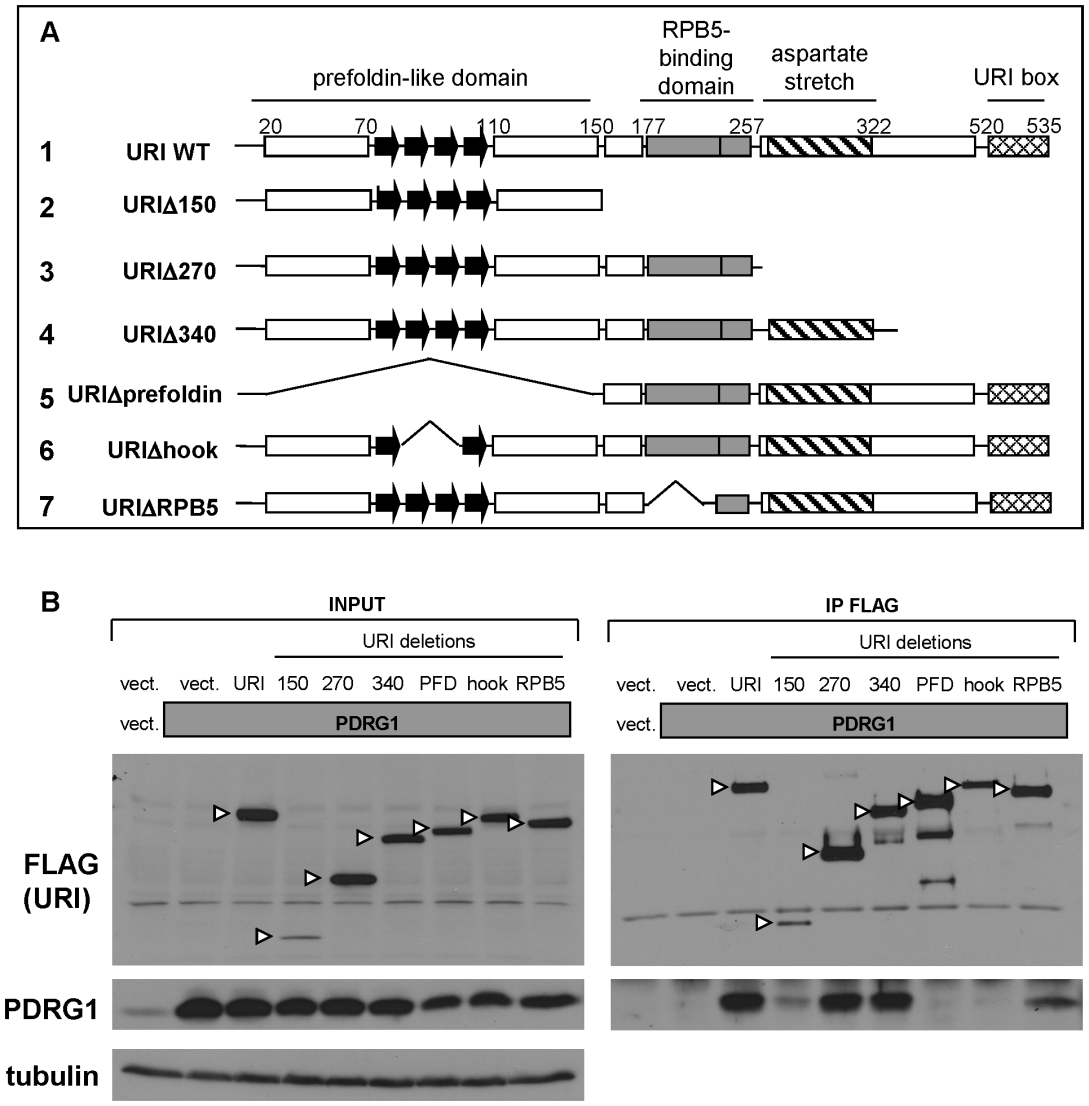
URI binds PDRG1 through the prefoldin-like domain. a) Schematic of the seven URI constructs used to map the domains of interaction between URI and PDRG1. The known URI domains are reported and the numbers on top indicate the corresponding amino-acid number. b) An empty vector (vect.), PDRG1 (gray box; 5 µg) and the different URI constructs were transfected into HEK293 cells (10 µg total DNA transfected). URI deleted of the prefoldin domain is labeled PFD. 48 hrs after transfection cells were lysed. Part of the lysate was used as INPUT (left panel) and another part (≈1 mg of proteins) was used to immunoprecipitate URI using FLAG antibodies. The immunocomplexes were analyzed by Western blot using the indicated antibodies. Arrowheads indicate the URI deletions/truncations.

The URI domain essential for the interaction with PDRG1 is not known. Because PDRG1 is a prefoldin-like protein similar to Art-27 we predicted that the prefoldin-like domain of URI is essential for interaction with PDRG1. Analysis of the interaction of deleted or truncated URI mutants (fig.4a) with overexpressed PDRG1 revealed that URI interacts with PDRG1 through the hook-shaped beta strands of the URI N-terminus prefoldin-like domain (fig.4b) which is also essential for the interaction with Art-27.

Overall, these results demonstrate that URI and PDRG1 interact with each other in prostate cells. Most likely URI, PDRG1, Art-27 and RPB5 are part of a common multiprotein complex in which URI has a pivotal role, because changes in URI expression induce either destabilization of the complex or increased expression of different members of the complex.

### Alteration of URI protein level does not affect RUVBL1 and RUBL2 transcription and protein stability

**Figure 5:**
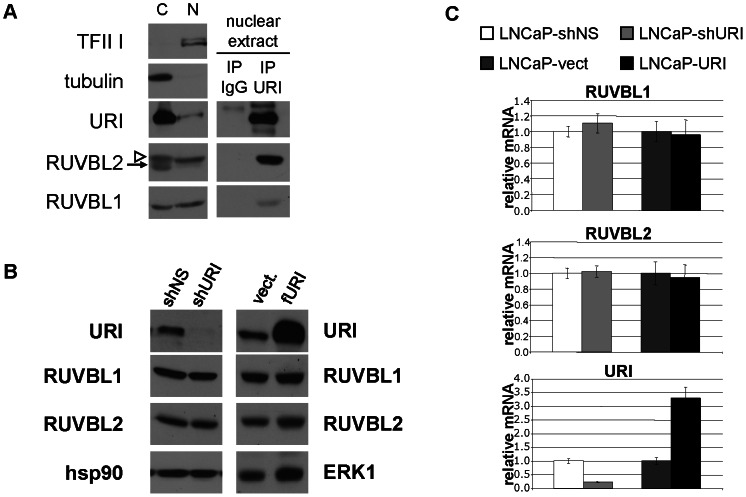
URI depletion or overexpression does not alter RUVBL1 and RUVBL2 protein levels. a) Cytoplasmic (C) and nuclear extracts (N) were isolated from LNCaP cells stably overexpressing URI. Nuclear extracts were used to immunoprecipitate URI and co-immunoprecipiatin of URI with RUVBL1 and RUVBL2 was analyzed by Western blot analysis. The white arrowhead indicates the RUVBL2 band while the arrows indicates a nonspecific band recognized by the RUVBL2 antibody. LNCaP cell lines stably expressing a non silencing shRNA (shNS), a shRNA against URI (shURI), an empty vector (vect.) or a construct encoding URI (URI) were lysed and the expression of RUVBL1 and RUVBL2 protein and mRNA was analyzed by Western blot analysis (b) and qPCR (c). hsp90 and ERK1 were used as loading controls.

Several MS analyses showed that the two ATP-dependent chromatin remodeling complexes RUVBL1 and RUVBL2 bind members of the R2TP/prefoldin-like complex [7], [8], [19]. Also, Izumi and colleagues recently validated the binding of URI to RUVBL1 and RUVBL2 by co-immunoprecipiation assays using Hela cell extracts [20] and demonstrated that RUVBL1, RUVBL2 and URI are part of a putative chaperone-regulatory complex that regulates and stabilizes the phosphatidylinositol 3-kinase-related protein kinases (PIKK) [21]. Interestingly, Pontin/RUVBL1 and Reptin/RUVBL2 have been shown to repress the transcription of several genes regulated by c-Myc, E2F1 and NF-kB [22]. Because of their biological relevance we decided to investigate if, as for other members of the R2TP/prefoldin-like complex, URI depletion or overexpression affected the cellular levels of the two RUVB-like proteins. We show that URI interacts with RUVBL1 and RUVBL2 in the nucleus of prostate cancer cells (fig.5a). However, analysis of RUVBL1 and RUVBL2 expression indicates that neither depletion nor overexpression of URI in prostate LNCaP cells affects mRNA expression (fig.5c) or protein levels (fig.5b) of RUVBL1 and RUVBL2. This may be explained by the fact that RUVBL1 and RUVBL2 are part of several important multi-protein complexes including the TRRAP/TIP60, the SRCAP and the hINO80 complexes [8]. Therefore, RUVBL1 and RUVBL2 can exist in complexes that do not contain URI, suggesting that RUVBL1 and RUVBL2 proteins may not be affected by altered URI levels since URI is just one of their many interacting proteins.

### URI shuttling between the cytoplasm and the nucleus is dependent on CRM1/XPO1-exportin

Our mass spectrometry and immunoprecipitation experiments suggest that URI is an essential protein for the stability of several members of the R2TP/prefoldin-like complex. Because this complex was shown to be important in the assembly of the RNA polymerase II complex in the cytoplasm [7] of the cells, URI probably has an important role in this process. Interestingly our mass spectrometry analysis also showed that the R2TP/prefoldin-like complex binds to URI in the nucleus of prostate cells suggesting that URI and the prefoldin-like complex may move with RNA pol II from the cytoplasm to the nucleus and vice versa.

**Figure 6:**
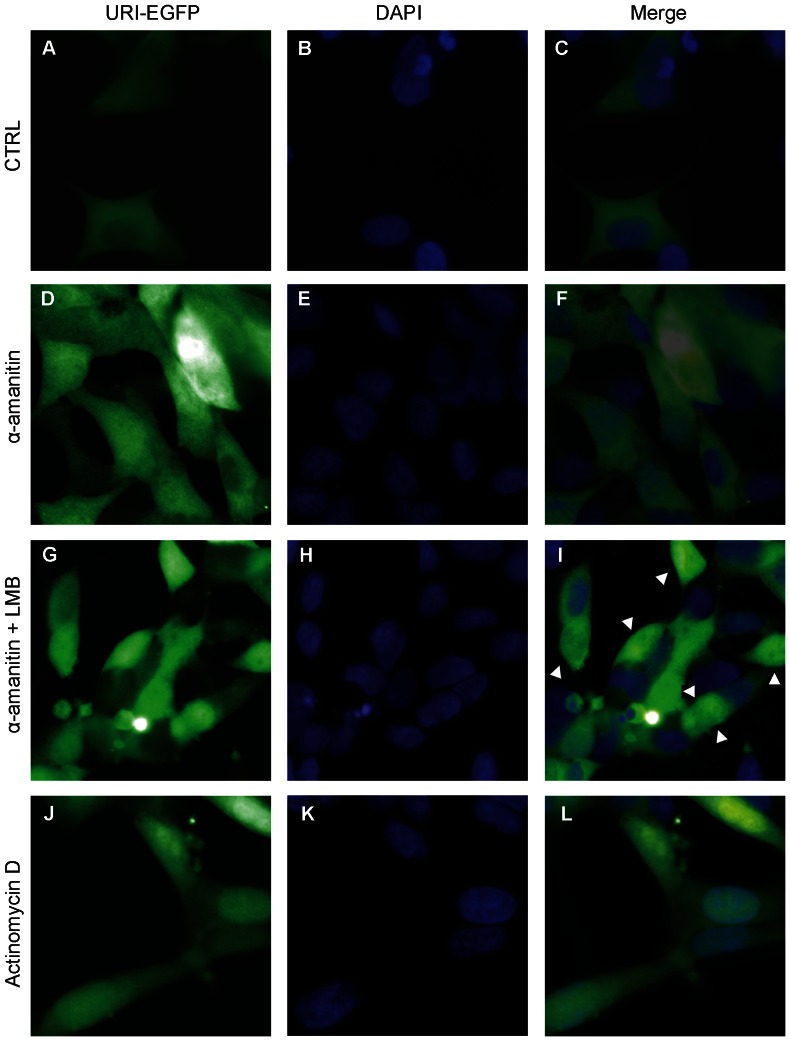
URI shuttling between the cytoplasm and the nucleus is dependent on CRM1/XPO1-exportin. LNCaP cells stably overexpressing an URI-EGFP fusion protein were used to visualize URI localization (a,b and c). Cells were treated with α-amanitin (d,e and f), α-amanitin + leptomycin B (g, h and i) or with actinomycin D (j–l). Cells were counterstained with DAPI (visualized as blue fluorescence). Single colors and merged pictures are presented.

To explore the role of URI in the R2TP/prefoldin-like complex and RNA pol II assembly we generated a LNCaP stable cell line over-expressing a URI protein fused to EGFP. This cell line was used to visualize URI movement upon treatment with compounds shown to affect the localization of pol II subunits. In particular, we used α-amanitin, which binds the large subunit of human RNA polymerase II (RPB1) and irreversibly stalls it on the DNA [23]. This results in transcriptional arrest and degradation of RPB1. Upon degradation of RPB1, RNA pol II disassembles and is exported into the cytoplasm through the CRM1/XPO1 exportin [24]. Leptomycin B (LMB) is a specific inhibitor of the CRM1 exportin that induces retention of the pol II subunit into the nucleus even after RPB1 degradation induced by α-amanitin [24]. Actinomycin D induces RNA pol II stalling on the DNA similar to α-amanitin, but it does not induce RPB1 degradation and disassembly of the RNA pol II complex [23]. In LNCaP-URI-EGFP cell lines, URI was mainly cytoplasmic (fig.6a–c). Upon treatment with α-amanitin, which induces RNA pol II stalling on the DNA, URI became more uniformly distributed between the cytoplasm and the nucleus suggesting that URI binds stalled RNA polymerase II accumulating in the nucleus (fig.6d–f). Treatment of the cells with α-amanitin and leptomycin B (LMB) induced increased nuclear localization and, in some cells URI became mainly nuclear (fig.6g–i, arrowheads). These data suggest that, like the RNA polymerase subunits, URI is exported out from the nucleus into the cytoplasm through the CRM1 exportin. In cells treated with actinomycin D (fig.6j–l) which induces RNA pol II stalling but not pol II disassembly URI became predominantly nuclear, suggesting tight binding of URI to RNA polymerase II even under conditions of stalled polymerase and transcriptional arrest.

Our data show that URI, probably in complex with Art-27, shuttles from the nucleus to the cytoplasm through the CRM1 exportin complex previously shown to mediate nuclear export of RNA polymerase subunits. Also nuclear URI increases in conditions in which pol II is stalled on the DNA suggesting binding of URI to the RNA polymerase complex and common mechanisms of nuclear export between URI and RNA polymerase subunits.

### Novel URI sites of phosphorylation or acetylation do not affect binding to RPB5 and Art-27

**Figure 7:**
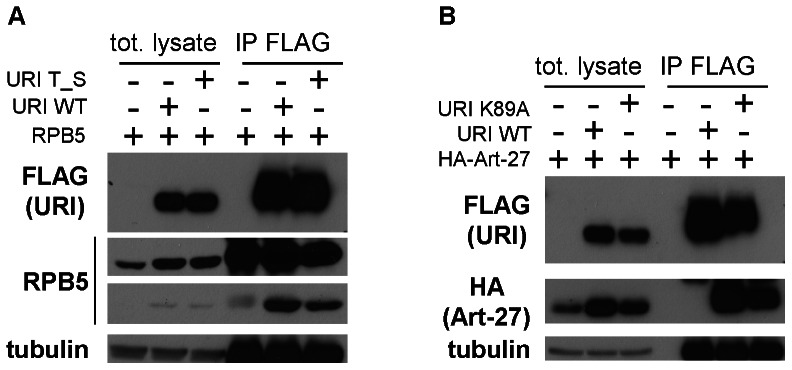
URI phosphorylation on T241, S243 and URI acetylation on K89 does not interfere with URI interaction with RPB5 and Art-27. 293 cells were transfected with a construct encoding RPB5 (a) or HA-Art-27 (b) and a construct encoding URI wild type (WT), URI T241A/S243A (URI T_S)(a) or URI K89A (b). FLAG-URI was then immunoprecipitated with FLAG antibodies and HA-Art-27 or RPB5 were analyzed by Western blotting using specific antibodies. Tubulin was used as loading control.

The phosphorylation and acetylation status of the immunoprecipitated FLAG-URI was also analyzed by mass spectrometry. This analysis revealed several sites of potential phosphorylation and acetylation of the URI protein. URI is phosphorylated on threonine 241 and serine 243. These residues are part of an interesting cluster (^241^TTSS^244^) shown to be phosphorylated in HeLa cells upon epidermal growth factor (EGF) stimulation [25], and predicted to be phosphorylated by casein kinase 2 (CK2). Phosphorylation of threonine 241 and serine 243 is also interesting because these amino acids are part of the accessory RPB5 binding domain. Other sites of potential phosphorylation of URI detected in our mass spectrometry analysis were serine 412 in the URI domain shown to be important for TFIIF interaction [26], and serines 442 and 449 located between TFIIF interaction domain and the URI box. Interestingly, serine 442 has been shown to be phosphorylated in the G1 phase of the cell cycle in a proteomic study aimed to identify mitosis-specific phosphorylation sites [27]. We also identified several potential sites of acetylation of URI. URI was acetylated on lysine 230, in the RPB5 accessory-binding domain, on lysine 89, in the hook of the prefoldin-like domain, and on lysine 466, between the TFIIF interaction domain and the URI box. A summary of URI candidate phosphorylation and acetylation sites identified in our analysis is reported in table 2. We also investigated if URI post-transcriptional modifications interfere with its interaction with other proteins of interest. In particular we tested if phosphorylation of URI on T241 and S243 interferes with the binding to RPB5 because these two residues reside in the RPB5 accessory-binding domain. We also investigated the effect of lysine 89 acetylation on the interaction of URI with Art-27 because amino acid 89 is part of the prefoldin-like domain hook that we and other groups showed to be essential for URI binding to Art-27. We generated constructs encoding URI T241A/S243A and URI K89A and expressed these constructs and wild type URI in 293 cells together with RPB5 or a HA tagged Art-27. Both the URI mutants interact with RPB5 or Art-27 similarly to wild type URI (fig.7a and b) indicating that URI T241/S243 phosphorylation does not interfere with RPB5 interaction and URI K89 acetylation does not interfere with Art-27 interaction. However, it is important to note that because the kinase and the cellular signaling that induces phosphorylation or acetylation of URI is not known, we cannot exclude the possibility that basal URI modification at these sites is not enough to observe differences in interaction between overexpressed URI WT and URI mutants with associated proteins. It is possible that unstimulated cells, used for mass spectrometry analysis and for the immunoprecipitation experiments presented in figure 7, have very low URI T241/S243 phosphorylation and URI K89 acetylation which were detected by mass spectrometry due to the high sensitivity of this technique. Further experiments that explore the role of URI phosphorylation and acetylation on these sites will need to be performed and will likely be of great biological interest considering the previously demonstrated importance of post-transcriptional modification for URI regulation and function [2], [5], [9].

**Table 2:** Identified sites of potential URI post-transcriptional modification.

Potential URI Modifications		
Amino acid	Domain	Type of modification
T241	RPB5 accessory binding domain	phosphorylation
S243	RPB5 accessory binding domain	phosphorylation
S412	TFIIF binding domain	phosphorylation
S449	C terminus	phosphorylation
S442	C terminus	phosphorylation
K230	RPB5 accessory binding domain	acetylation
K89	prefoldin-like domain	acetylation
K466	C terminus	acetylaton

Potential URI post-transcriptional modifications detected by our mass spectrometry analysis. Phosphorylation and acetylation sites are reported.

It is possible that URI phosphorylation may be an important step that triggers the cascade of events that leads to active transcription by pol II. According to this hypothesis URI would act as an important hub of regulation that “translates” extracellular signaling into particular landscapes of transcription activation.

## Discussion

**Figure 8:**
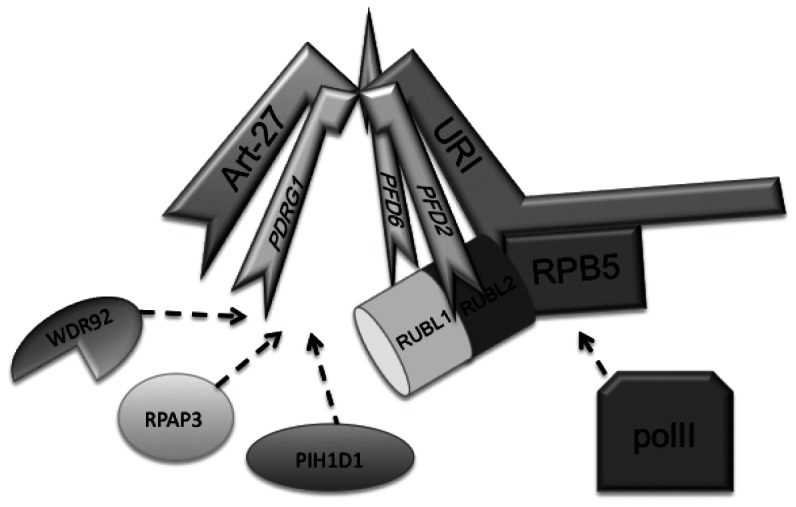
Model for the interaction of the R2TP/prefoldin-like components. The prefoldin-like components of the complex (URI, Art-27, PDRG1 and presumably PFD2 and PFD6) interact through the “hook” of their prefoldin-like domain. URI also binds RPB5 with a.a. 177–224 which most likely connects the whole prefoldin-like complex to the RNA polymerase II.

URI was previously shown to be a scaffold protein coordinating the interaction of proteins involved in transcription such as TFIIF [26], the Paf-1 complex [28] and the RNA polymerase II itself through the binding to the RPB5 subunit [1]. URI was identified as a RNA polymerase interacting protein able to repress transcription and we also show that this repressive function holds true in the context of androgen receptor mediated transcription [5]. Despite the several proteins identified as URI interactors, including the androgen receptor corepressor Art-27, the molecular mechanism by which URI is able to repress transcription is unknown. To shed light into the nuclear function of URI, we performed a mass spectrometry analysis to identify proteins that specifically bind URI in the nucleus of prostate cells. This analysis confirmed the previously published interaction of URI with RPB5 and Art-27 and further characterizes an intriguing chaperone-like complex suggested to mediate the RNA polymerase II complex assembly in the cytoplasm of the cells. This complex was named R2TP/prefoldin-like complex because of the shared subunits with the yeast R2TP complex and the homology of several components with the prefoldin proteins. We showed that URI is a key component of the R2TP/prefoldin-like complex because depletion of URI induces degradation of several components of the complex such as Art-27 and PDRG1, suggesting destabilization of the entire complex upon URI loss. Also, URI overexpression induces increased expression and stability of Art-27, PDRG1 and RPB5. The crystal structure of the R2TP/prefoldin-like complex is today unknown. Our data as well as the previously reported “jellyfish-like” structure of the hexameric prefoldin complex [29] suggest that the prefoldin-like proteins of the R2TP/prefoldin-like complex (URI, Art-27, PDRG1, PFD2 and PFD6) probably all interact with each other through the beta strands of their prefoldin-like domain (fig.8).

Interestingly, experiments that examine URI cytoplasmic/nuclear translocation using a stable cell line expressing a URI-EGFP fusion protein, show that URI shuttles from the nucleus to the cytoplasm in a CRM1 dependent manner, a mechanism also used by RNA pol II subunits to translocate into the cytoplasm and be recycled for the formation of new pol II complexes. Our data suggest that URI and Art-27 bind RPB5, and presumably the RNA polymerase II as part of the R2TP/prefoldin-like complex in the cytoplasm and are shuttled in and out of the nucleus following the RNA polymerase II complex.

Our mass spectrometry analysis also revealed several sites of known and potential phosphorylation and acetylation of URI, some of which have been also reported before [25], [27]. We showed that phosphorylation of the sites within the RPB5 binding domain (T241 and S243) and acetylation of the site within the domain involved in Art-27 binding (K89) does not affect binding of URI to either RPB5 or Art-27. Further studies need to be done to better elucidate the role of post-transcriptional modification of these specific sites. It is intriguing to hypothesize that phosphorylation or acetylation of URI affects the transcriptional repression activity of URI, thereby inducing changes in the URI multiprotein complex.

Overall, our work adds insight to the understanding of the R2TP/prefoldin-like complex, several members of which are intimately connected to URI for their stability. Moreover, we presented evidence suggesting that URI moves from the nucleus to the cytoplasm through the CRM1/XPO1 exportin, elucidating, at least in part, a previously unknown mechanism of nuclear translocation for the URI/Art-27 complex. This finding may shed light on the aberrant nuclear exclusion of Art-27 found in recurrent prostate cancer [30]. Overall, the data presented here underscore the need for additional studies elucidating the nature and function of the R2TP/prefoldin-like complex, an enigmatic protein complex that may have strong implications in transcriptional modulation by all three RNA polymerases.
